# Extreme Accelerations During Earthquakes Caused by Elastic Flapping Effect

**DOI:** 10.1038/s41598-018-37716-y

**Published:** 2019-02-04

**Authors:** Hiroyuki Goto, Yoshihiro Kaneko, John Young, Hamish Avery, Len Damiano

**Affiliations:** 1 Disaster Prevention Research Institute, Kyoto University, Uji, Kyoto 6110011 Japan; 2 GNS Science, Lower Hutt, 5040 New Zealand; 3 Canterbury Seismic Instruments Ltd, Christchurch, 8041 New Zealand

## Abstract

Accurate recording of large, earthquake-induced ground shaking is critical for our understanding of earthquake physics as well as seismic hazard assessment. Extremely large accelerations with the peak value of 3.2 times the gravity acceleration were recorded at seismic station WTMC located in northern South Island of New Zealand during the recent magnitude 7.8 Kaikoura earthquake. However, the mechanisms responsible for the generation of such large accelerations are not well understood. Here we use numerical simulations to examine a range of physical models that can reproduce the observed characteristics of the acceleration record. We find that the record of the asymmetric, vertical accelerations, also observed during a magnitude 6.3 earthquake, can be explained by a flapping effect, that is, the local, elastic bouncing of a foundation slab on which the sensor is installed. Our results suggest that the extremely large accelerations recorded at seismic station WTMC do not reflect the actual ground shaking, but were caused by a local, system response around the sensor. Our finding has important implications for both the evaluation of future seismic hazard based on the waveform records of the Kaikoura earthquake and the installation methodology of strong-motion seismometers in all earthquake prone countries.

## Introduction

Ground motion records are footprints of ground shaking during earthquakes and are widely utilized in seismology and earthquake engineering. For example, the cause of damage to buildings and infrastructures under large ground shaking has been examined on the basis of the ground motion records^1,2^. Key structures such as hospitals, dams and power plants are often designed based on the analysis of past records of large ground motions to prevent catastrophic damage due to future earthquakes^3,4^. Ground motion records are also used to assess landslide and liquefaction hazard^5–8^. Every application stands on an assumption that ground motion records correctly reflect the actual ground shaking during an earthquake. If the records were contaminated with a local, system response, i.e., the response of a structure at which the sensor is installed, it should not be directly used for other applications. Especially for occasions when extremely large ground motion is recorded^9–12^, identifying factors that may contaminate the actual ground shaking is of critical importance for disaster reduction for future earthquakes.

**Figure 1:**
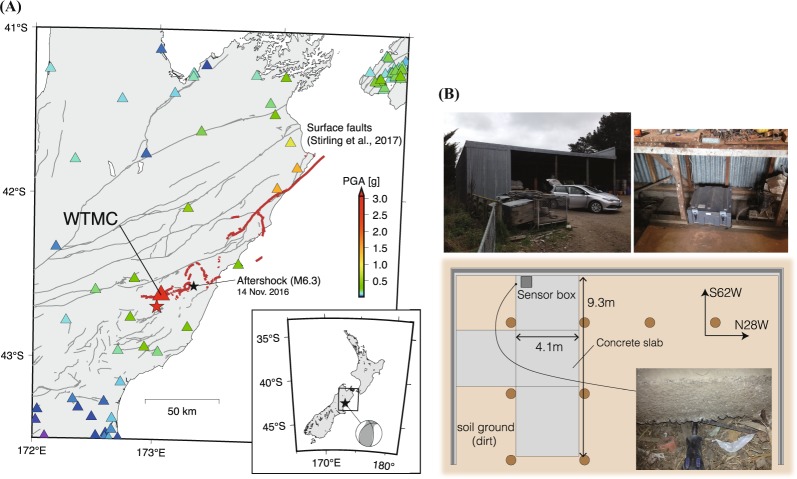
Location and installation of seismic station WTMC. (**A**) Map of peak ground accelerations (PGA) during the 13th November 2016 Mw7.8 Kaikoura earthquake. Largest PGA (3.23 g) was recorded at seismic station WTMC. Red solid lines are surface traces of faults ruptured during the Kaikoura earthquake^35^, and gray lines are active faults in this region^36^. **(B)** Plan view of station WTMC. An acceleration seismometer (contained in gray box) is situated on a concrete slab in a farm shed. Some minor gaps between the concrete slab and soil ground were found by our site investigation.

The 13th November 2016 Mw7.8 Kaikoura earthquake resulted in high ground motion records at several seismic stations spanning more than 6000 km^2^ of northern South Island of New Zealand^13^. Among them, an anomalously large peak acceleration of 3.23 g in the vector sum of three components was recorded at seismic station WTMC (S42.6211°, E173.0535°), which is located in Waiau, Northern Canterbury in the vicinity of faults that ruptured during the earthquake (Fig. 1)^13–15^. This acceleration was considered as the second highest peak ground acceleration (PGA) ever observed for any earthquake in the world; the highest PGA of 4.10 g was recorded at seismic station IWTH25 during the 2008 Mw6.9 Iwate-Miyagi earthquake, Japan^16^. At seismic station WTMC, an acceleration seismometer (CUSP-3C sensor) is situated on a concrete slab in a farm shed (Fig. 1). Site investigation immediately after the Kaikoura earthquake concluded that the seismometer at seismic station WTMC was functioning correctly and the sensor box was firmly attached to the concrete slab^13^.

**Figure 2:**
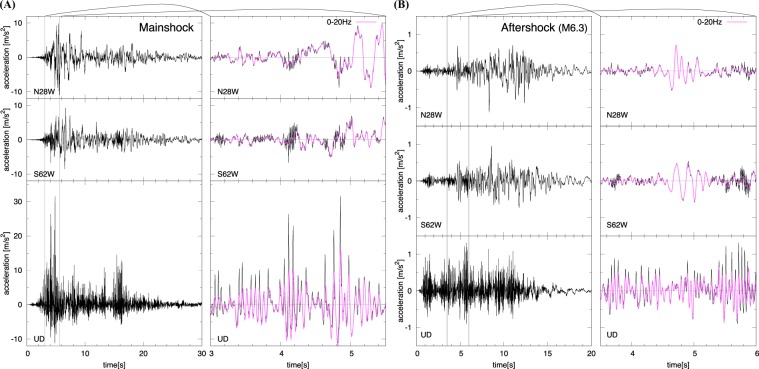
Acceleration records at seismic station WTMC. (**A**) the mainshock of the Kaikoura earthquake and **(B)** a M6.3 aftershock that occurred on 14th Nov (see Fig. 1A). The asymmetric, vertical accelerations (AsVA) are reduced in the low-pass filtered (<20 Hz) waveforms (in pink). AsVA also appear in the aftershock waveforms despite the much smaller PGA (~0.1 g).

Acceleration data at seismic station WTMC during the Kaikoura mainshock show unique characteristics (Fig. 2). The vertical motion clearly exhibits an asymmetry with upward and downward peak accelerations around 3 g and −1g, respectively (Fig. 2). The dominant frequency in the up-down motions is 13.9 Hz, whereas the sharp upward peaks are made of higher frequency components (30–34 Hz). Large upward accelerations are accompanied by high-frequency oscillations (80–85 Hz) in the horizontal components (Fig. 2). Both the large asymmetric, vertical accelerations and horizontal high-frequency oscillations disappear when the data are low-pass-filtered at 20 Hz (Fig. 2). Interestingly, one of the major aftershocks (M6.3, Fig. 1) also shows similar acceleration characteristics at seismic station WTMC despite the much smaller PGA (~0.1 g) (Fig. 2).

The mechanism responsible for large asymmetric, vertical accelerations (AsVA) has been attributed to the decoupling of near-surface materials referred to as a ‘trampoline’ effect^16–19^, or a rocking effect^20,21^. The trampoline effect occurs when a rebound force causes a large upward acceleration and a downward motion is limited at the gravitational acceleration (1 g) due to the bouncing of a deformable soil^16^. In contrast, the rocking effect is characterized by upward accelerations induced by large horizontal motion when the structural basement underneath the seismic sensor impacts on the ground surface^20^. The important difference between the trampoline and rocking effects is that the former is caused by actual ground shaking, whereas the latter is due to the interaction of a structure and soil.

The AsVA recorded at station WTMC are not consistent with the trampoline or rocking effect. The trampoline effect would involve a free-fall of the soil ground caused by the vertical acceleration exceeding 1 g, and hence cannot explain relatively small (~0.1 g) AsVA during the M6.3 aftershock (Fig. 2). In addition, vertically eccentric structures needed to induce the rocking effect were not found at station WTMC (see Fig. 1). Therefore another mechanism must be responsible for the origin of the extremely large AsVA.

## Results

**Figure 3:**
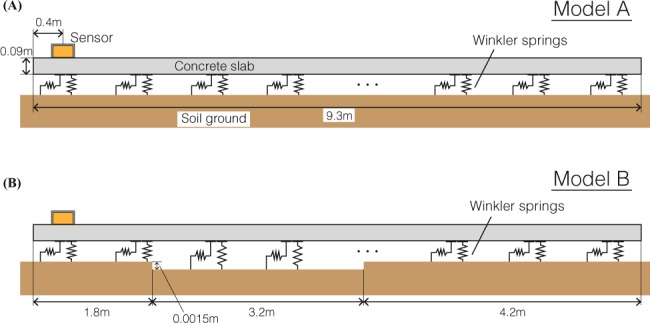
Two schematic models that may explain the characteristics of acceleration records at seismic station WTMC. (**A**) Model A is based on the bouncing of a deformable mass. **(B)** Model B is proposed in this study to explain the characteristics of acceleration records for both the Kaikoura mainshock and a M6.3 aftershock. The horizontal dimension corresponds to the S62°W direction shown in Fig. 1B.

We aim to reproduce the characteristics of the acceleration data at seismic station WTMC using finite-element modeling of dynamic interactions between the concrete foundation slab and underlying soil (Methods). Based on the concept of the bouncing of a deformable mass^16^, we develop a model that consists of the foundation slab sitting on a flat soil surface, with the slab lengths and thickness as those measured at station WTMC (Model A in Fig. 3). During earthquake ground shaking, the reaction force from the soil to the slab is represented by Winkler springs - a system of identical but mutually independent, linear elastic springs. Vertical springs do not act in tension (i.e., the spring stiffness is set to be zero) when the initial weight is released during the bouncing of the foundation slab. This allows the separation of the slab from the soil and can lead to the bouncing of a deformable mass. Low-pass filtered (<20 Hz) acceleration waveforms (pink curves in Fig. 2) are assumed as input ground motions and are applied to Model A.

**Figure 4:**
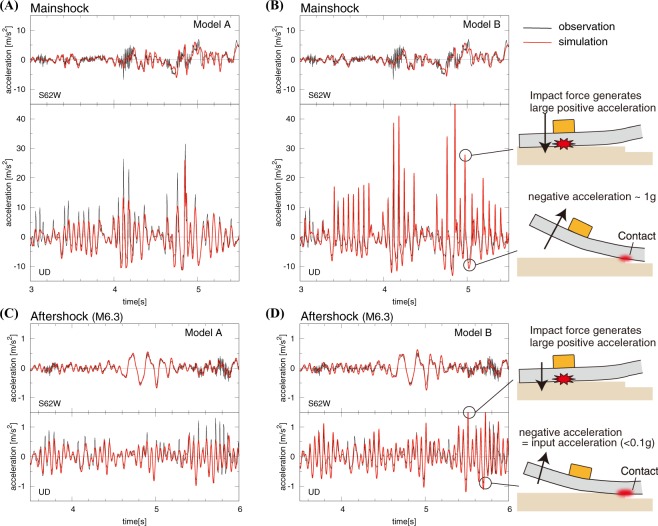
Simulation results for the Kaikoura mainshock and a M6.3 aftershock. Simulated vertical and horizontal accelerations (in red) at the location of the sensor are compared against the data (in black) recorded at seismic station WTMC. Schematic figures illustrate the mechanism of AsVA due to elastic flapping in Model B.

Simulations of the WTMC sensor motion based on Model A correctly reproduce the observed characteristics of the accelerations for the Kaikoura mainshock (Fig. 4A; Movie S1) including the AsVA and high-frequency horizontal oscillations. However, of the range of parameters we explored (Methods; Fig. S2), none of the cases for Model A generate sufficiently large AsVA and high-frequency horizontal oscillations for the M6.3 aftershock (Fig. 4). The system of Model A is horizontally uniform, and thus separation between the foundation slab and soil occurs along the entire surface simultaneously during the bouncing of the slab (Movie S1). More than 1 g upward acceleration is required for the bouncing of the slab, because all the weight must be released from the springs. Therefore, in Model A, AsVA cannot be generated during small vertical accelerations induced by the M6.3 aftershock.

To explain both the mainshock and aftershock records, we propose a new model consisting of the foundation slab and an irregular contact surface between the slab and underlying soil (Model B in Fig. 3). Such an irregular contact surface, which would have been created by differential settlement of the soil or soil erosion over time, was confirmed by our site investigation; some minor gaps (<1 cm) between the concrete slab and soil ground were found, as shown by the screwdriver in the bottom right photo of Fig. 1B. In addition, a full contact surface is not commonly found in nature, as suggested by the theory of tribology^22,23^. In contrast to Model A, the irregular contact surface allows local elastic bouncing of the concrete slab during earthquake ground shaking.

Simulations based on Model B reproduce the main characteristics of the recorded accelerations at station WTMC during both the mainshock and aftershock (Fig. 4; Methods). The irregular contact surface enables the separation of the slab from the soil locally, even during weaker ground motions (~0.1 g), resulting in enhanced positive accelerations at the location of the sensor (Fig. 4). The elastic bouncing occurs in the form of the flapping of a smaller section of the slab on which the sensor is located, resulting in AsVA and high-frequency horizontal oscillations (Movie S2). Unlike Model A, some contacts between the slab and soil always remain even during the large mainshock motions (>1 g). During the weaker aftershock motion (<1 g), the negative acceleration is controlled by the input ground motion (<1 g), while the positive acceleration is enhanced when the slab impacts the soil ground locally, and hence the AsVA occurs (Fig. 4; Movie S3).

The horizontal high-frequency oscillations in these models are accompanied with the occurrence of AsVA, although their amplitudes are slightly smaller than the data (Fig. 4). In these models, the horizontal high-frequency oscillations are produced when the horizontally and vertically moving slab impacts the soil (Fig. 4). At that instance, the horizontal motion is momentarily restricted by the contact friction, resulting in high-frequency oscillations. The amplitude of the horizontal high-frequency oscillations depends on the vertical stress and is larger when the positive vertical acceleration is higher. Therefore the concurrent occurrence of the AsVA and horizontal high-frequency oscillations would have resulted from the coupling of the vertical and horizontal motions via elastic flapping of the slab.

**Figure 5:**
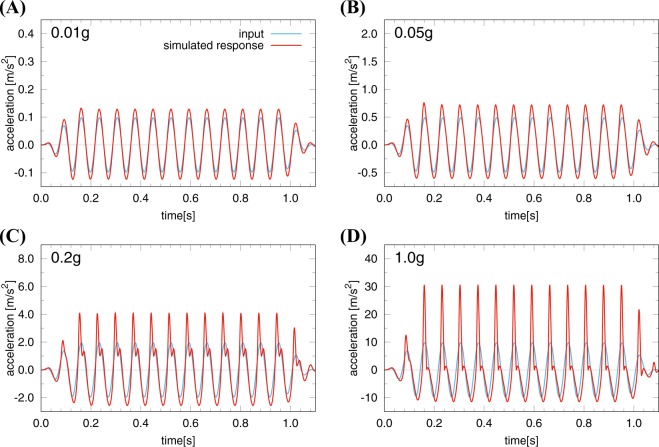
Simulated responses of Model B under sinusoidal input ground motions with a variety of amplitudes: (**A**) 0.01 g, (**B**) 0.05 g, (**C**) 0.2 g, and (**D**) 1.0 g. Simulated responses in Model B (in red) are compared to the input accelerations (in cyan). The ratios of the response to the input-motion amplitude are 1.35, 1.54, 2.10, and 3.13, respectively. The flapping effect amplifies when the input ground is larger. Pulses in the positive (or upward) direction are spiky, showing the generation of higher frequency components that were not present in the input motions.

To further analyze the response of slab-soil interactions in Model B, we simulate vertical acceleration responses under artificial, input motions that are simple sinusoidal accelerations with a variety of amplitudes (Fig. 5). The excitation frequency is 13.9 Hz, which is the dominant frequency of the vertical component of the mainshock record. The amplitudes of the input accelerations are 0.01 g, 0.05 g, 0.2 g, and 1.0 g, respectively. The input excitation continues 1.1 seconds and is tapered from and to zero during the first and last two-cyclic motions. The responses are examined using Model B (Fig. 3B).

Simulated responses under the sinusoidal input motions further support our findings. Under weak motions (0.01 and 0.05 g), the simulated responses exhibit sinusoidal accelerations similar to the input motions (Fig. 5). In contrast, AsVAs are generated under large input motions (0.2 and 1.0 g) (Fig. 5). When the input motion is large (>0.1 g), the simulated responses are spiky and characterized by higher-frequency, asymmetrical accelerations (Fig. 5), consistent with the mainshock waveform characterized by the high-frequency (30–34 Hz) spikes. In addition, larger input motions enhance the AsVAs (Fig. 5), consistent with the mainshock and aftershock records at station WTMC.

## Discussion

Although the motions of the foundation slab in Model B are locally similar to the rocking effect, the mechanism is fundamentally different. The elastic bouncing of the slab, which we refer to as a flapping effect, is induced by vertical motion through a system with variation in the horizontal direction, e.g., the foundation slab sitting on an irregular surface. In addition, we found no evidence for the excitation of the rocking of the slab as there were no vertically eccentric structures around the sensor at station WTMC (see Fig. 1). Our analysis suggests that soil-slab interactions characterized by the flapping effect led to the AsVA and extreme acceleration at station WTMC. Note that while similar processes of soil-structure interactions under earthquake-induced ground motions have been discussed in literatures^24–27^, we demonstrate that the flapping effect can indeed explain the observed AsVA and extreme acceleration.

Our modeling results suggest that large AsVA (the peak value of 3.23 g) in the WTMC records do not reflect the actual ground motions generated by the Kaikoura earthquake waves. The large AsVA must have been involved in a local, system response around the sensor. While the actual ground motions would still have been large (~1.2 g), as inferred from the low-pass-filtered waveforms (Fig. 2), the extremely large peak acceleration of 3.23 g was likely caused by the particular site condition at station WTMC. Our finding has important implications for evaluating future seismic hazard based on the waveform records of the Kaikoura earthquake. If the records were assumed to reflect the actual ground shaking, there would be significant impact on the design of buildings and key structures.

Several important simplifications are made in our model. First, we assume that soil-slab interactions can be represented by linear elastic springs in compression without considering soil nonlinearity. The effect of soil nonlinearity likely becomes important near the edge of gaps, which may reduce the impact of the slab onto the soil^28,29^. To address this issue, we further consider Model B accounting for a simplified form of soil plasticity (Methods; Fig. S5). The modeling results show that, while the soil plasticity reduces the amplitude of the accelerations to some degree, the AsVA and extreme acceleration still occur regardless of soil nonlinearity (Methods; Fig. S5). In addition, 3-D response accounting for the longitudinal direction of the shed (Fig. 1B) may enhance the flapping effect at the corner of the slab. Although more realistic models considering soil nonlinearity and 3-D effect would be needed for quantitative understanding, our model presented here is simple enough to reveal the principal mechanism of the AsVA recorded at station WTMC.

The recordings of AsVA during other large earthquakes were documented at seismic stations elsewhere^19^, where the instruments are also situated on concrete slabs. While we have not yet investigated the site conditions of these seismic stations, the flapping effect proposed here might also explain the origin of AsVA reported previously, as the elastic flapping of a concrete slab can occur over a wide range of physical parameters when the input ground motions exceed ~0.05 g (Methods).

Our results have important implications for how strong-motion sensors in New Zealand and elsewhere are installed to ensure the recordings of actual ground motions, rather than soil-slab interactions. Since station WTMC is situated in a typical farm shed and the flapping effect is less obvious during weaker motions (<0.1 g) caused by more frequent, smaller earthquakes (M < 6), it is not straightforward to identify *a priori* potential soil-slab interactions at seismic stations. To prevent similar soil-slab interactions at other strong-motion sensor sites, we suggest the following strategies; (1) a foundation slab needs to be firmly attached to the soil ground, for example, using piles or anchors, (2) slab properties (thickness, stiffness) are designed to minimize the effect of elastic bouncing, or the flapping effect, or (3) a sensor is placed directly onto soil ground (e.g., outside a shed). Ensuring the recording of actual ground motions by seismic sensors is critical for proper assessment of ground motion intensity during past earthquakes and disaster mitigation for future destructive earthquakes.

## Methods

### Description of seismic station WTMC

Seismic station WTMC is located in Waiau, Northern Canterbury, New Zealand. The sensor is situated on a concrete slab in a farm shed (see Fig. 1), which was constructed on gentle sloping ground dipping to the south (Fig. S1). The land was likely leveled by filling soil beneath the concrete slab, and hence the soil is thicker in the southern side where the sensor was installed. It appears that there are no reinforcing bars connecting the slab and ring foundation (see the photo in Fig. S1B), indicating that the slab is free to rub against the ring foundation during earthquake shaking.

The acceleration seismometer (CUSP-3C sensor, yellow box in Fig. S1) at station WTMC weights 3.1 kg has a dimension of 280 mm by 260 mm by 130 mm (height). The sensor is firmly bolted to a gray sensor box, which weights 36.7 kg (including cables, batteries, and the sensor) and has a dimension of 550 mm by 550 mm by 250 mm (height). The sensor box is firmly attached to the concrete slab in the depth to 30 mm via four anchors. Site investigation immediately after the Kaikoura mainshock concluded that the sensor, sensor box and concrete slab are firmly attached to one another. The acceleration waveforms are recorded by a sampling frequency of 200 Hz and filtered with gain and phase responses shown in Fig. S1D. The sensor response of up to 85 Hz is ensured, and therefore, high-frequency oscillations in the horizontal component at 80–85 Hz (Fig. 2A) would be an actual signal, although any higher frequency component would have been filtered out.

At the time of the Kaikoura mainshock, packed fertilizer bags of various weights (10–250 kg) were stacked on the concrete slab within a few meters from the sensor. Some of these fertilizer bags fell onto the concrete slab during the mainshock. However, the moving and bouncing of the packed bags cannot explain the AsVA. Packed bags impacting onto the slab should generate large negative accelerations, which is opposite from the recorded AsVA. From our site investigation after the Kaikoura earthquake, no cracks were found on the concrete slab. Hence the slab would have behaved elastically during the strong ground shaking.

### Numerical modeling

In our numerical simulations, the concrete slab is modeled by finite elements on the 2-D profile (N62°E-S62°W), which is the narrow direction of the shed. The length and thickness of the slab are 9.3 m and 0.09 m, respectively. Young’s modulus and density are set to be 30000 kN/m^2^ and 2400 kg/m^3^, respectively, representing the physical properties of a standard concrete. The slab is discretized into 0.1 m width of beam elements with three degrees of freedom per node: horizontal, vertical translation and rotational components. Reaction force from the soil is modeled by Winkler springs. The vertical springs do not act in tension, which represents soil-slab separation. The horizontal springs represent frictional force, whose maximum value is constrained by static friction proportional to the normal force. When the horizontal force exceeds the maximum static friction (static friction coefficient by normal force), horizontal sliding occurs. These springs are connected to all the nodes.

Self-gravity deformation is calculated prior to the ground motion excitation. This process sets the initial condition of the model, e.g. initial deformation of the elastic slab, initial weights acting on each spring, etc. Dynamic responses for the mainshock and M6.3 aftershock are simulated, followed by the self-gravity calculation. Low-pass filtered (<20 Hz) records at station WTMC (see Fig. 2) are assumed as the actual input ground motion, and high-frequency components are induced by the dynamic response of soil-slab interactions. Vertical and horizontal accelerations at the sensor location on the slab are the model output and compared with acceleration time series recorded at station WTMC.

### Parameter study

Unknown model parameters unconstrained by our site investigation include the location and clearance of soil-slab gaps and soil spring stiffness. Hence we simulate the dynamic response for several plausible models with a variety of parameter sets.

AsVA observed during the M6.3 aftershock is one of the important features in constraining the model parameters. We set a spatially-uniform vertical spring coefficient, *kv*, to be a parameter of Models A and B, and assess the reproducibility of the AsVA. The parameter range of *kv* is related to the range of plausible S-wave velocity of the soil through the formulation by^30^. The static friction coefficient is fixed to be 0.25 for both models. Appearance of AsVA is quantified by the following values^19^:

1 $${F}_{{\rm{As}}}=\frac{{a}_{max}+{a}_{min}}{\max ({a}_{max},\,-\,{a}_{min})},$$

where *a*_max_ and *a*_min_ are the maximum and minimum values of vertical accelerations within two cycles of dominant periods, 0.144 s. The values indicate tendency for the asymmetry, and purely symmetric records give *F*_As_ = 0. The averaged values of *F*_As_ over 4–5 s for the original and low-pass filtered records of the mainshock are 0.492 and 0.104, respectively. The averaged values of *F*_As_ over 5–6 s for the aftershock records are 0.354 and 0.147, respectively.

Figure S2 shows modeled AsVA with a variety of vertical spring coefficients. For the mainshock, both Models A and B can generate AsVA consistent with the original records in terms of averaged values of *F*_As_, but the larger *kv* (>40 MPa/m) cannot excite large enough asymmetry for Model A. In contrast, the aftershock record tightly constrains the model parameter space, as not all the models can generate large enough AsVA for the aftershock. Only Model B with *kv* ~ 30–40 MPa/m can achieve the observed *F*_As_. This is because Model A explains the asymmetry via the bouncing of a deformable mass, and the small ground motion (~0.1 g) is insufficient to move up the entire slab in the air simultaneously. This behavior does not change in a range *kv* > 120 MPa/m.

Gaps beneath the slab affect the appearance of AsVA. Since the detailed location and dimension of gaps beneath the slab are not available, we simply characterize the location and width of the gap parameterized by gap origin and width in Model B, as shown in Fig. S3, and examine the modeling results with a variety of the parameters. The vertical spring and static friction coefficients are set to be 35 MPa/m and 0.25, respectively. We find that the simulation results are largely insensitive to the values of static friction coefficient. The gap depth is 0.0015 m. The appearance of AsVA is quantified by *F*_As_ as in the previous paragraph, and the accuracy of each model is measured by the misfit between the peak ground accelerations defined by

2 $$PG{A}_{{\rm{residual}}}=\frac{|{a}_{max}|-|{\bar{a}}_{max}|}{|{\bar{a}}_{max}|},$$

where ${a}_{max}$ and ${\bar{a}}_{max}$ are simulated and observed peak ground acceleration in the vertical component, respectively. Smaller *PGA*_residual_ means the model is better matched to the observed record in terms of the PGA.

Figure S3 shows *F*_As_ and *PGA*_residual_ values for the mainshock and the M6.3 aftershock. Large *F*_As_ are simulated in large gap origin and width cases for the mainshock, whereas large *F*_As_ can be generated with a certain parameter set for the aftershock (Fig. S3). Because *F*_As_ is constrained by the appearance of AsVA, the gap origin and width should be larger than 1.0 m and 2.5 m, respectively. Smaller *PGA*_residuals_ are obtained in the case of the gap origin of about 0.8 m and the gap width of 2.4 m for the mainshock, and in the case of the gap width of 3.2 m for the aftershock. The best parameter set in terms of reproducibility for the aftershock records is indicated by ‘x’ symbol in Fig. S3 and is shown as Model B in Fig. 3. Gap widths smaller than 3.2 m may be slightly better to explain the mainshock record; however, these cases are not consistent with the aftershock record. The minor differences of inferred gap dimensions between the mainshock and aftershock may be caused by the assumption of linear elastic springs. The elastic bouncing under large motion would result in a dynamic change of contact areas, and soil nonlinearity not accounted for by our modeling may significantly affect the dynamic response at the edge of the gaps.

### Other aftershock records

Figure S4 shows observed records and modeling results for three other seismic events (Event #1–3), using a parameter set of Model B. WTMC records are available for these events, which are selected from the aftershocks of the Kaikoura earthquake. PGAs of each record are less than 0.01 g. The simulated and observed waveforms are compared in a time window around the P-wave arrival because the maximum vertical acceleration for each event was recorded within this time window.

For these seismic events, AsVA are not observed (Fig. S4). This suggests that AsVA at WTMC only appear when the input vertical acceleration exceeds about 0.05 g, and are unrelated to any particular time. Our modeling results can explain these observations well in that Model B also does not generate AsVA during these seismic events. Separation between the slab and the soil surface is needed to generate the enhanced positive large accelerations and hence AsVA. Model B simulations suggest that the threshold of an input motion level required to cause the separation via elastic flapping effect is about 0.05 g, consistent with these observed records (Fig. S4).

### Effect of soil plasticity on simulated response

The mechanisms responsible for the AsVA observed at station WTMC are discussed so far on the basis of elastic soil springs and frictional sliding at the contact between the foundation slab and soil. At the same time, natural soil likely behaves plastically under large loading. Hence we explore the effect of soil plasticity on the response of the slab using the set-up of Model B accounting for soil plasticity.

Soil plasticity is generally described by the effects of both shear and compression. Since the shear behavior has been previously modeled as drag springs at a soil-structure interface^31^, which is similar to the concept of frictional sliding assumed in our model, we ignore the plastic shear behavior of the soil and account for only the plastic compression behavior, referred to as vertical plasticity. In our model, vertical plasticity is represented by the concept of critical state line (CSL) describing the relation between volumetric change and confining stress on the critical state^32^. We assume plasticity parameters based on widely used Toyoura sand^33,34^ and calculate the CSL. Past load, which sets the initial condition, is assumed to be 1.2 times the self-gravity. When the past load is much greater than the self-gravity, the model becomes purely elastic, whereas the past load of smaller than the self-gravity is unrealistic. The stress is a linear function of the spring force *kv* until it reaches the CSL. Once the stress reaches the CSL, it follows along the CSL.

Figure S5 compares the results with and without the vertical plasticity of soil. Due the plastic response, the maximum amplitude of simulated vertical accelerations decreases by 16.6 percent compared to the purely elastic counterpart (Fig. S5). However, AsVAs still present in the elasto-plastic model for both the mainshock and M6.3 aftershock (Fig. S5). Hystereses in the horizontal and vertical components underneath the sensor and the edge of a gap are shown in Fig. S5. The vertical hysteresis shows clear differences between the purely-elastic and elasto-plastic models. In the elasto-plastic model, the stress follows along the CSL under large loading and returns to an elastic response under unloading (Fig. S5). As expected, the horizontal hysteresis follows frictional behavior for both models (Fig. S5). These results suggest that nonlinearity of soil under the mainshock leads to quantitatively different responses while the AsVA and extreme acceleration caused by the flapping effect would have occurred regardless of soil nonlinearity.

## Supplementary information

Supplementary Information

Movie S1

Movie S2

Movie S3

## Data Availability

Original WTMC records are available from Strong Motion Data Products in GeoNet (https://www.geonet.org.nz/data/types/strong_motion).
