# Supplementary material for: Extreme Accelerations During Earthquakes Caused by Elastic Flapping Effect
Source: Sci Rep. 2019 Feb 4;9:1117. doi: 10.1038/s41598-018-37716-y (PMC6361895; doi:10.1038/s41598-018-37716-y)
Supplement: Supplementary file 1 — Supplementary Information [file 41598_2018_37716_MOESM1_ESM.pdf]

Supplementary Information

Extreme Accelerations During Earthquakes Caused by Elastic Flapping Effect

Hiroyuki Goto[1]*, Yoshihiro Kaneko[2], John Young[2], Hamish Avery[3], Len Damiano[3]

[1] Disaster Prevention Research Institute, Kyoto University, Uji, Kyoto, 6110011, Japan
[2] GNS Science, Lower Hutt, 5040, New Zealand
[3] Canterbury Seismic Instruments Ltd, Christchurch, 8041, New Zealand
* Corresponding author, Email: goto@catfish.dpri.kyoto-u.ac.jp

Contents

Fig. S1. Schematic figure of seismic station WTMC and cross section diagram around the sensor and sensor box at seismic station WTMC.
Fig. S2. Appearance of AsVA simulated from a variety set of vertical spring coefficients.
Fig. S3. Appearance of AsVA and PGA residuals simulated from a variety set of gap parameters.
Fig. S4. Simulation results for three aftershock events.
Fig. S5. Simulated responses of Model B accounting for soil plasticity.

Movie S1. Simulation movie of Model A for the mainshock
Movie S2. Simulation movie of Model B for the mainshock
Movie S3. Simulation movie of Model B for the aftershock

Captions

Fig. S1. Schematic figure of seismic station WTMC and cross section diagram around the sensor and sensor box at seismic station WTMC, (B) N62°E -S62°W and (C) N28°W-S28°E directions. (D) Filter gain and phase of CUSP-3C sensor.

Fig. S2. Appearance of AsVA (F_{As}) simulated from a variety set of vertical spring coefficients. (A) Model A and (B) Model B for the mainshock, and (C) Model A and (D) Model B for the aftershock. For the Model B simulations, the dimension and location of the gaps are fixed to the ones shown in Fig. 3.

Fig. S3. Appearance of AsVA (F_{As}) and PGA residuals simulated from a variety set of gap parameters on the basis of Model B. (A) F_{As} and (B) PGA_{residual} for the mainshock and (C) F_{As} and (D) PGA_{residual} for the aftershock. Symbols (X) shown in each panel indicate a parameter set with the gap origin at 1.8 m and the gap width of 3.2 m, corresponding to Model B shown in Fig. 3B.

Fig. S4. Simulation results for three aftershock events; 22:54 13th Nov. (Event #1), 06:47 14th Nov. (Event #2), and 19:17, 14th Nov. (Event #3) (UTC). Simulated results by Model B (in red) are compared to the observed records (in black) in horizontal (S62°W) and vertical components in a section around the P-wave arrival.

Fig. S5. Simulated responses of Model B accounting for soil plasticity. Simulated acceleration responses, and hysteresis loops beneath the sensor and the gap edge are shown for (A) the mainshock and (B) M6.3 aftershock. Critical state line (CSL, black line) is indicated in the hysteresis plots. Compared to the elastic responses, Model B accounting for soil plasticity reduces the amplitude of the vertical accelerations by 16.6 and 6.8 percent for the mainshock and M6.3 aftershock, respectively; however, as in the elastic case, AsVA is still generated.

Movie S1. Simulation of the Kaikoura mainshock motions in Model A. Vertical, dynamic deformation is enhanced 1000 times for visualization purpose. Top left panel is a close-up display around the sensor (green circle) shown in the bottom panel. Brown circles indicate contacts between the foundation slab and soil surface. The separation of the foundation slab and soil surface is illustrated by the disappearance of the brown circles. Top right panel shows input (pink line) and simulated response accelerations (red line) in vertical component.

Movie S2. Simulation of the Kaikoura mainshock motions in Model B. Vertical, dynamic deformation is enhanced 200 times for visualization purpose. Top left panel is a close-up display around the sensor (green circle) shown in the bottom panel. Brown circles indicate contacts between the foundation slab and soil surface. Top right panel shows input (pink line) and simulated response accelerations (red line) in vertical component.

Movie S3. Simulation of M6.3 aftershock motions in Model B. Vertical, dynamic deformation is enhanced 4000 times for visualization purpose. Top left panel is a close-up display around the sensor (green circle) shown in the bottom panel. Brown circles indicate contacts between the foundation slab and soil surface. Top right panel shows input (pink line) and simulated response accelerations (red line) in vertical component.

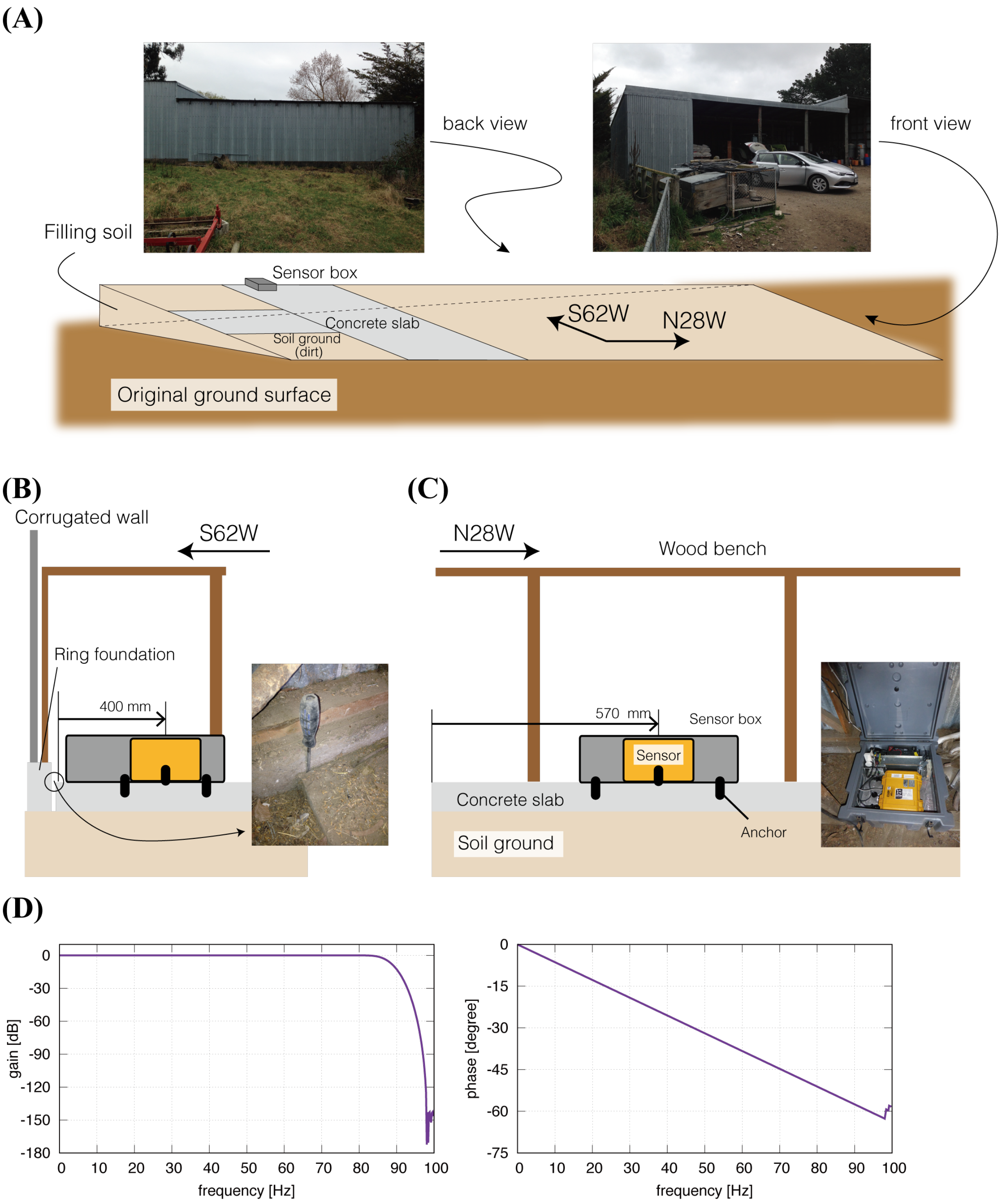

Fig. S1. Schematic figure of seismic station WTMC and cross section diagram around the sensor and sensor box at seismic station WTMC, (B) N62°E -S62°W and (C) N28°W-S28°E directions. (D) Filter gain and phase of CUSP-3C sensor.

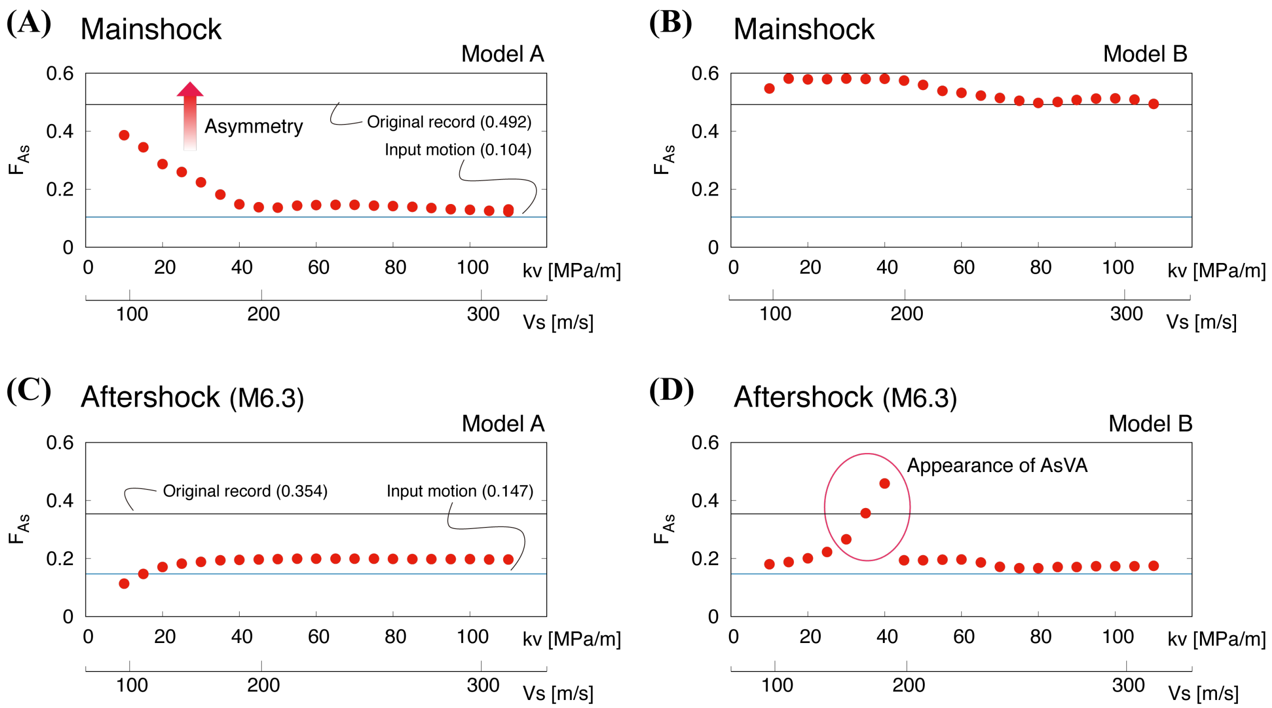

Fig. S2. Appearance of AsVA (F_{As}) simulated from a variety set of vertical spring coefficients. (A) Model A and (B) Model B for the mainshock, and (C) Model A and (D) Model B for the aftershock. For the Model B simulations, the dimension and location of the gaps are fixed to the ones shown in Fig. 3.

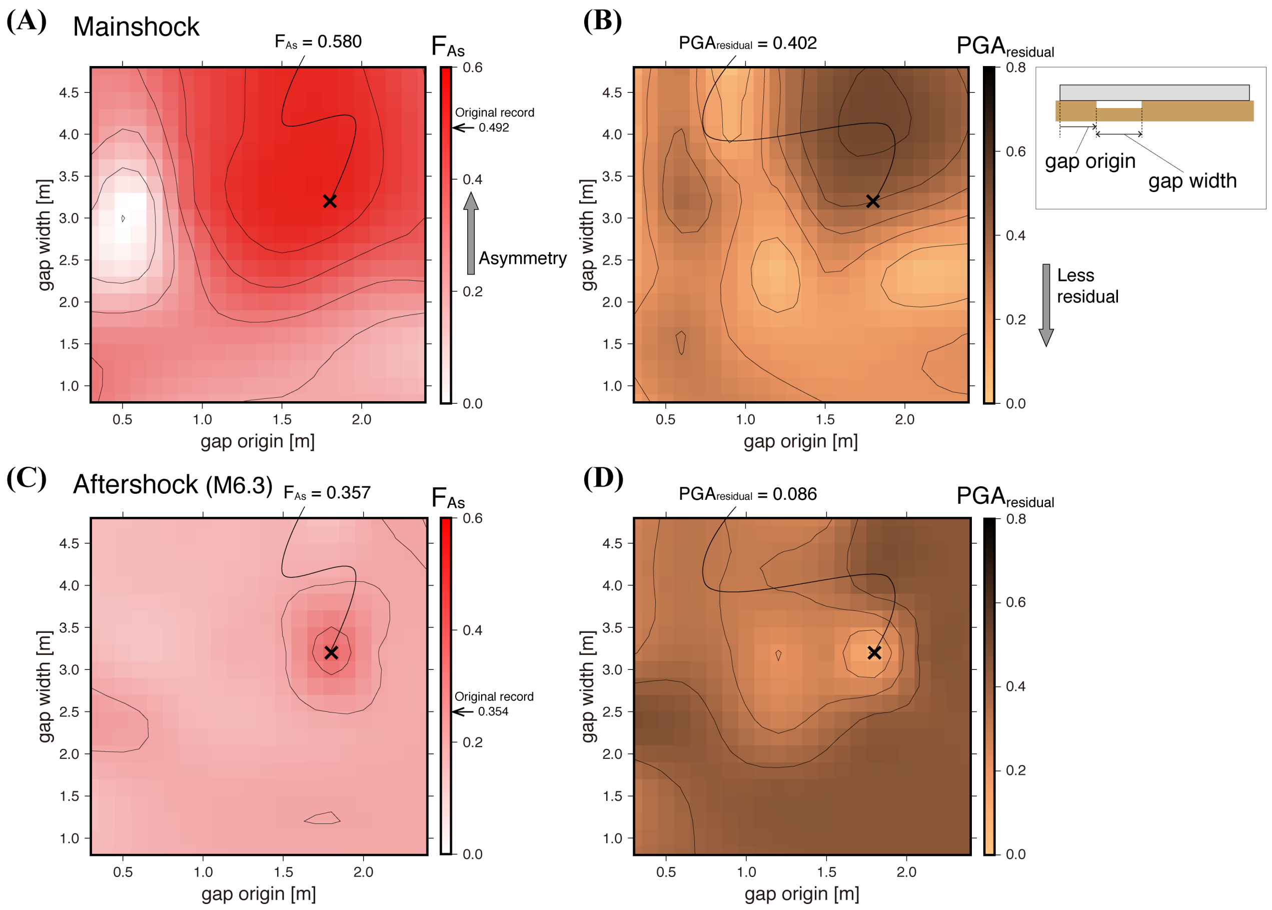

Fig. S3. Appearance of AsVA (F_{As}) and PGA residuals simulated from a variety set of gap parameters on the basis of Model B. (A) F_{As} and (B) $PGA_{residual}$ for the mainshock and (C) F_{As} and (D) $PGA_{residual}$ for the aftershock. Symbols (X) shown in each panel indicate a parameter set with the gap origin at 1.8 m and the gap width of 3.2 m, corresponding to Model B shown in Fig. 3B.

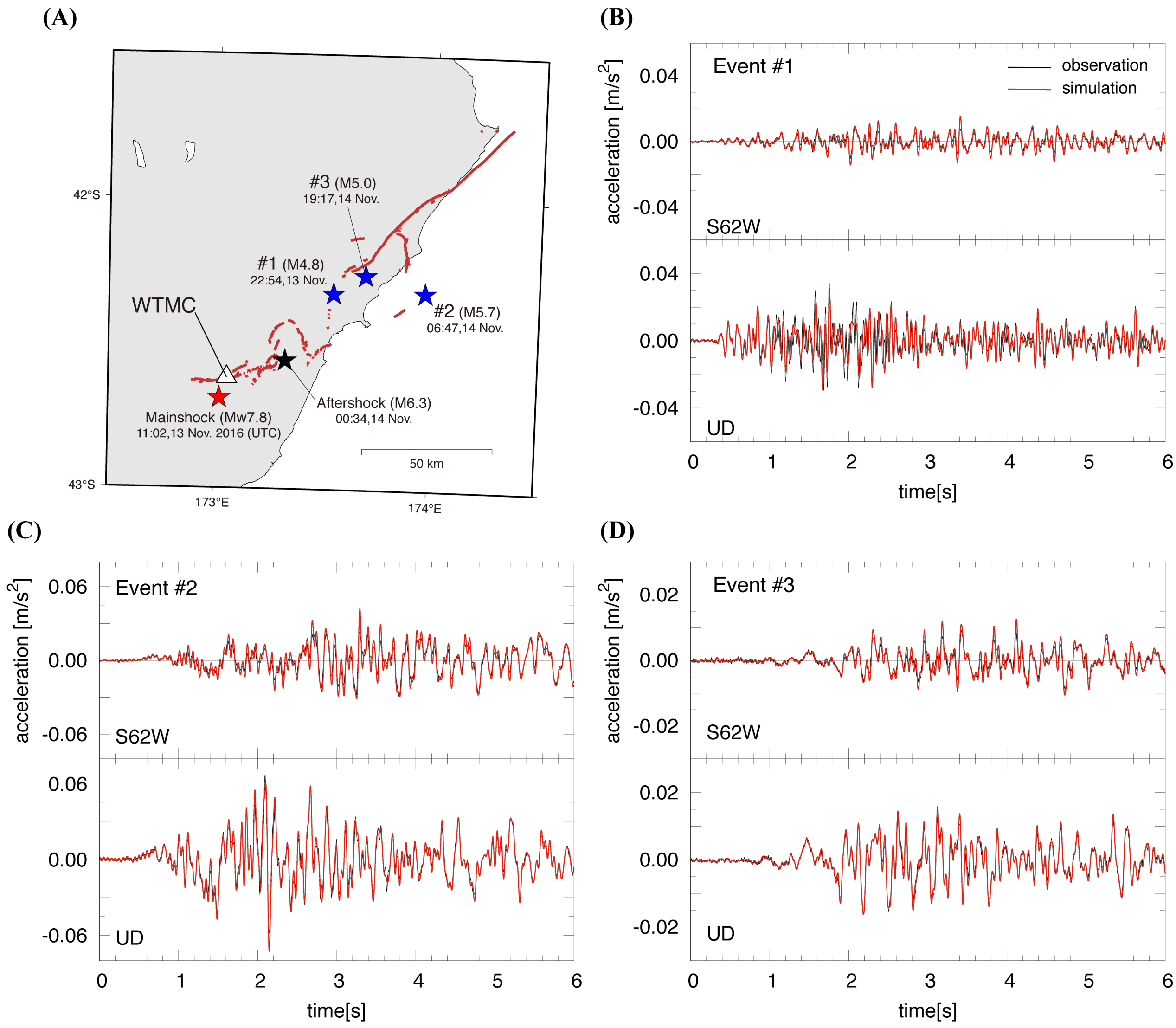

Fig. S4. Simulation results for three aftershock events; 22:54 13th Nov. (Event #1), 06:47 14th Nov. (Event #2), and 19:17, 14th Nov. (Event #3) (UTC). Simulated results by Model B (in red) are compared to the observed records (in black) in horizontal (S62°W) and vertical components in a section around the P-wave arrival.

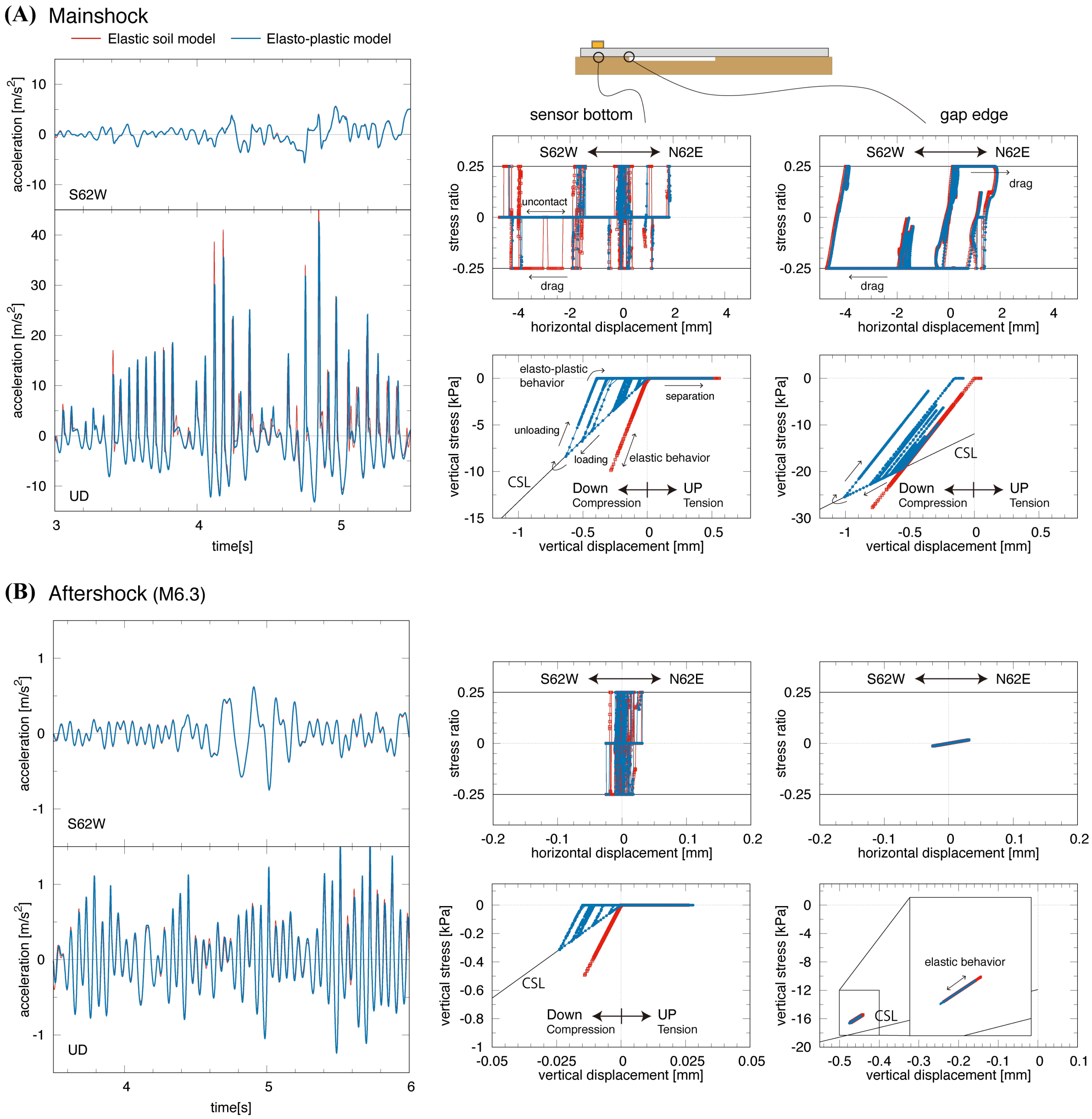

Fig. S5. Simulated responses of Model B accounting for soil plasticity. Simulated acceleration responses, and hysteresis loops beneath the sensor and the gap edge are shown for (A) the mainshock and (B) M6.3 aftershock. Critical state line (CSL, black line) is indicated in the hysteresis plots. Compared to the elastic responses, Model B accounting for soil plasticity reduces the amplitude of the vertical accelerations by 16.6 and 6.8 percent for the mainshock and M6.3 aftershock, respectively; however, as in the elastic case, AsVA is still generated.
